# Supplementary material for: Crests, troughs, and plateaus: Using story theory to explore the experiences of adolescents and young people living with HIV in Kampala, Uganda
Source: PLoS One. 2026 Mar 25;21(3):e0345499. doi: 10.1371/journal.pone.0345499 (PMC13016292; doi:10.1371/journal.pone.0345499)

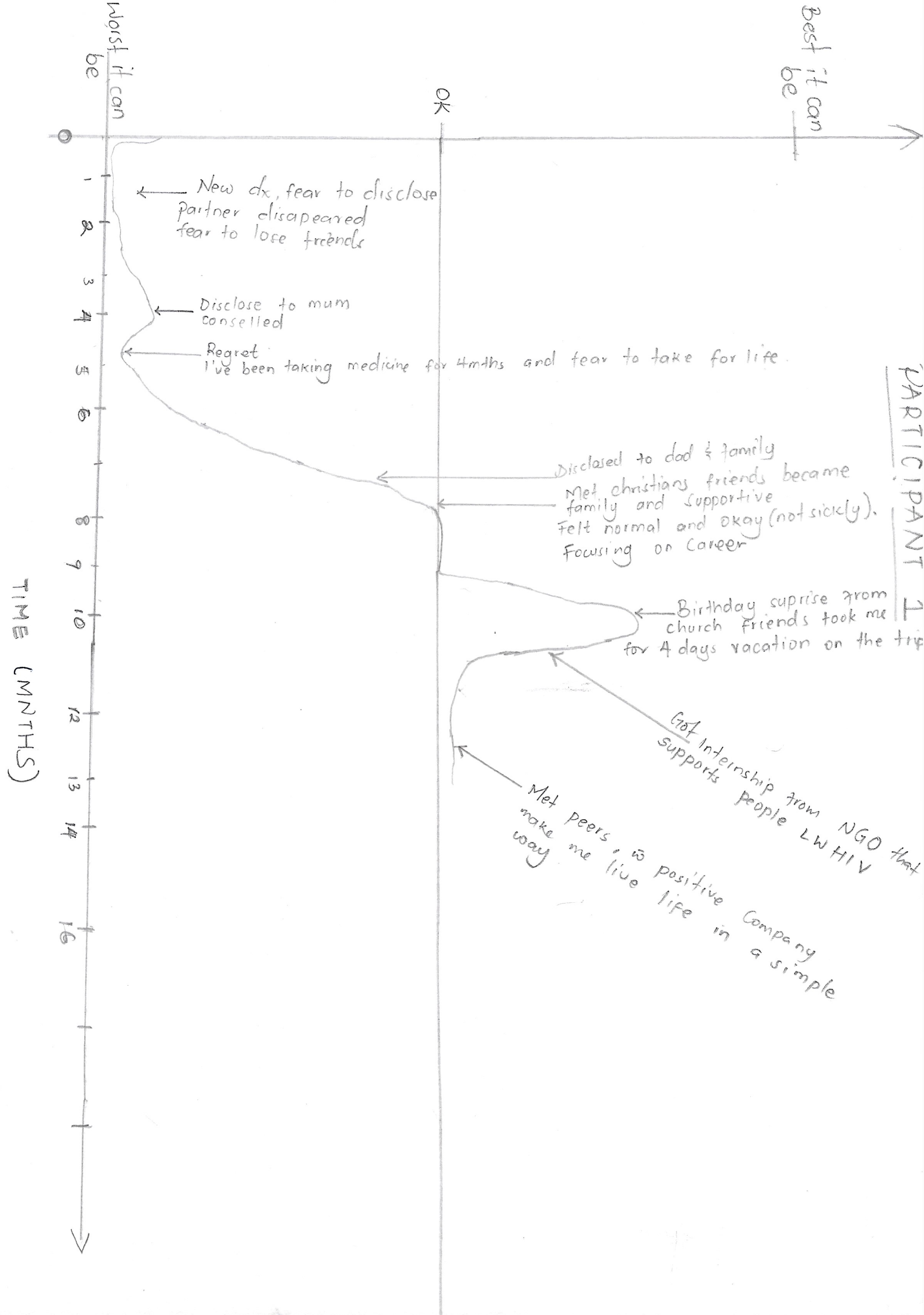
PARTICIPANT 1
Worst it can be
OK
Best it can be
0
TIME (MNTHS)
1
2
3
4
5
6
8
9
10
12
13
14
16
New dx, fear to disclose
Partner disapeared
fear to lose friends
Disclose to mum
consellled
Regret
I've been taking medicine for 4mths and fear to take for life.
Disclosed to dad & family
Met christians friends became family and supportive
Felt normal and okay (not sickly).
Focusing on Career
Birthday suprise from church friends took me for 4 days vacation on the [illegible]
Got Internship from NGO that supports people LWHIV
Met peers, & positive company make me live life in a simple way

PARTICIPANT 2

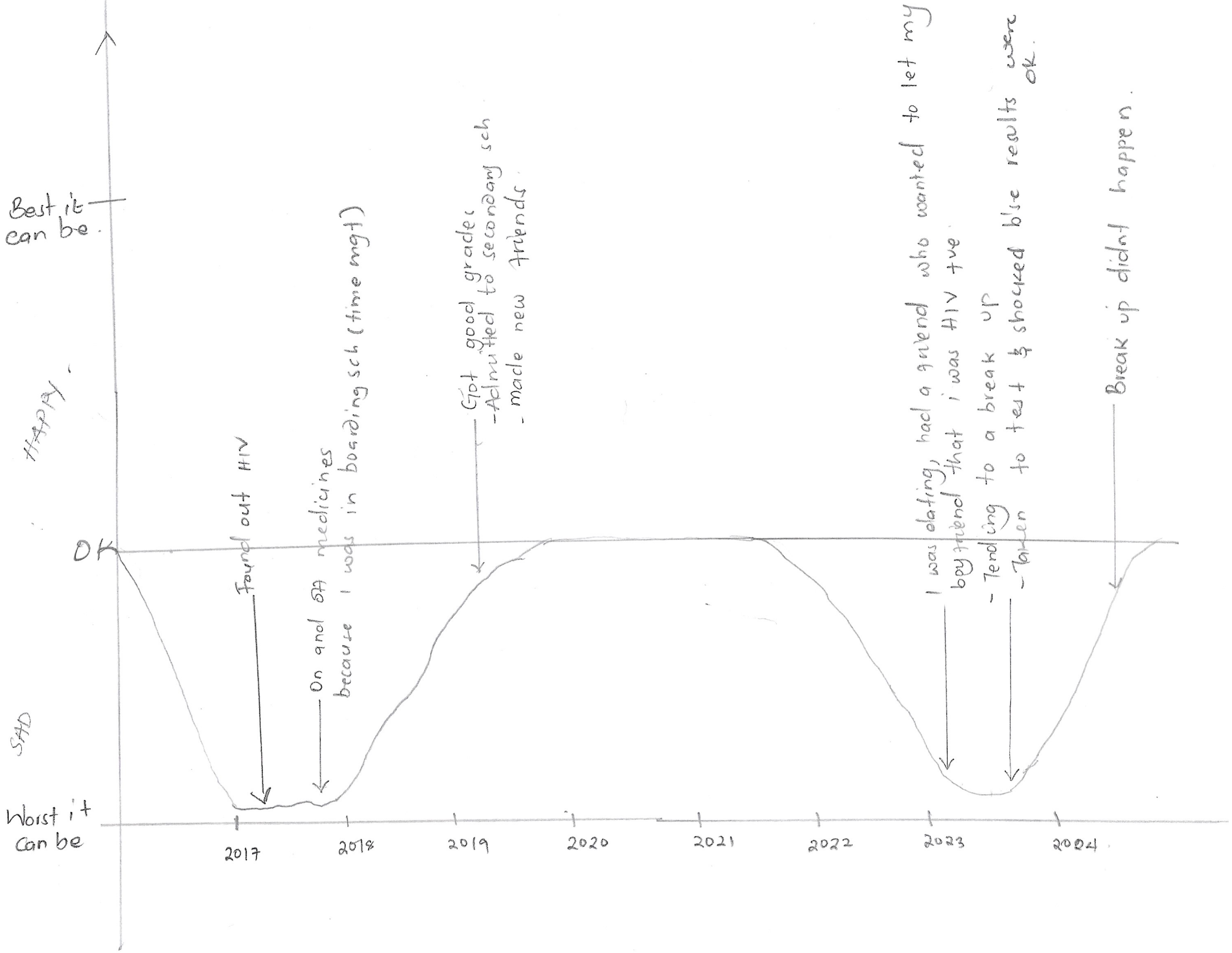

PARTICIPANT 3

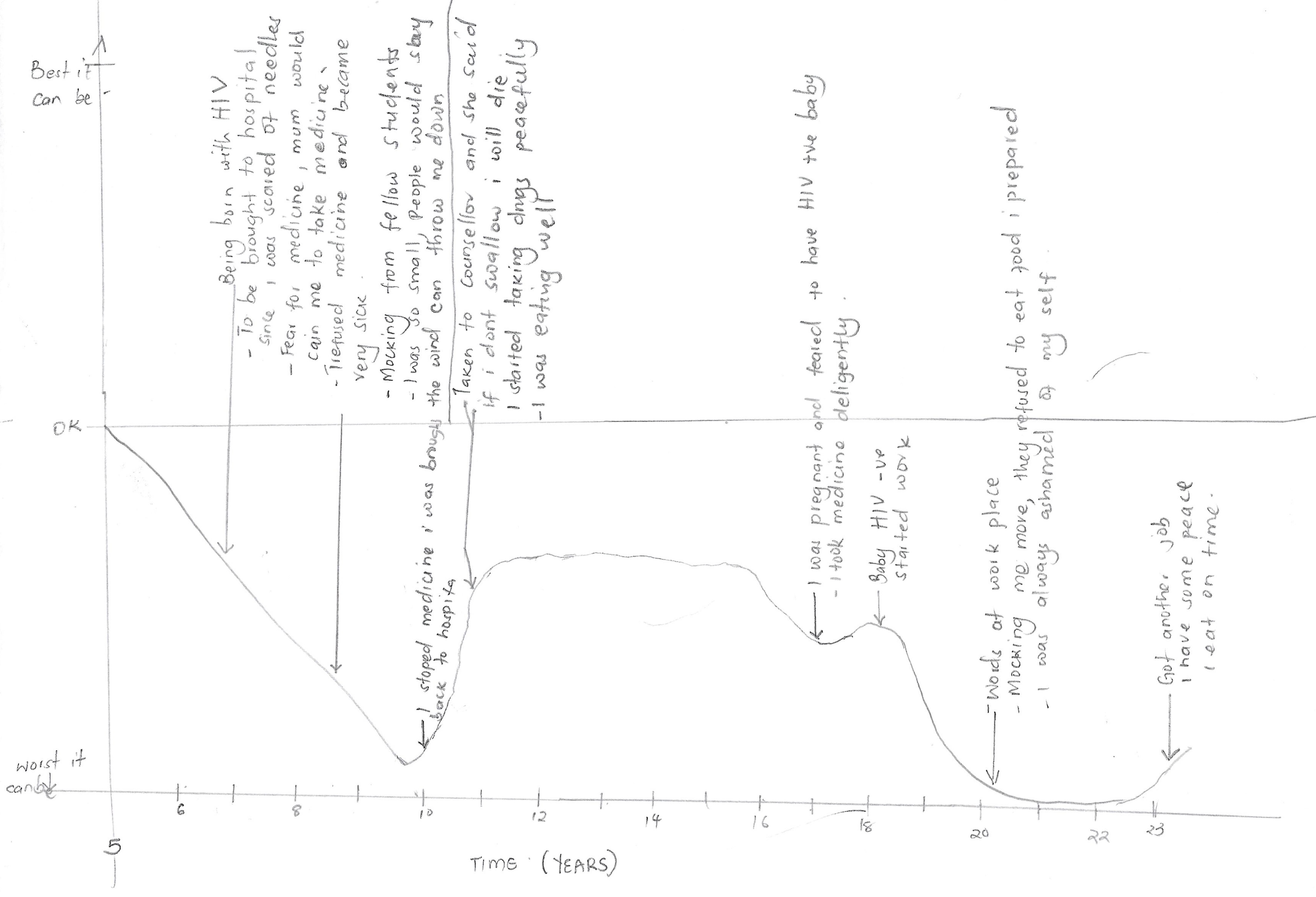

PARTICIPANT 4

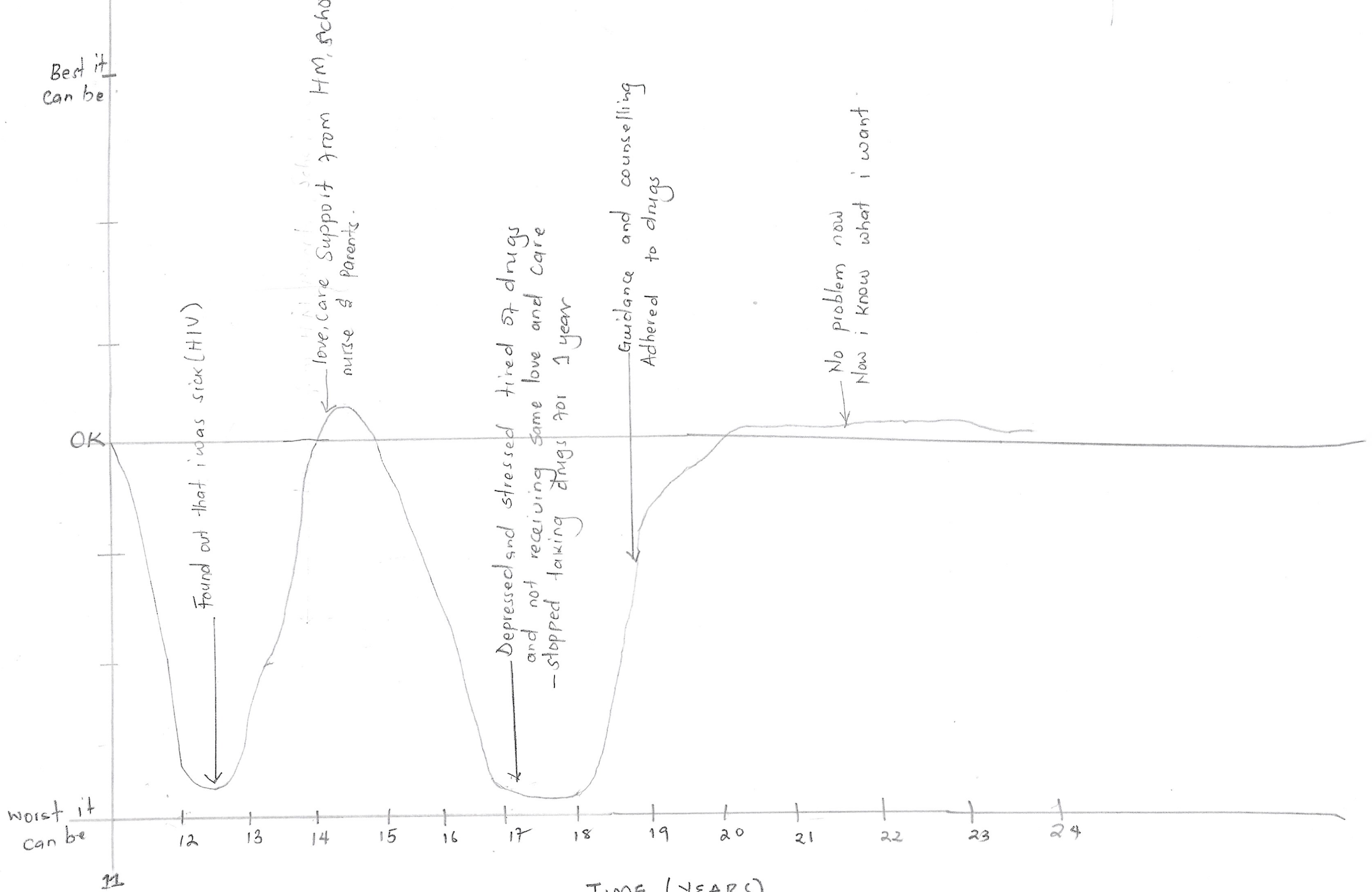

PARTICIPANT 5

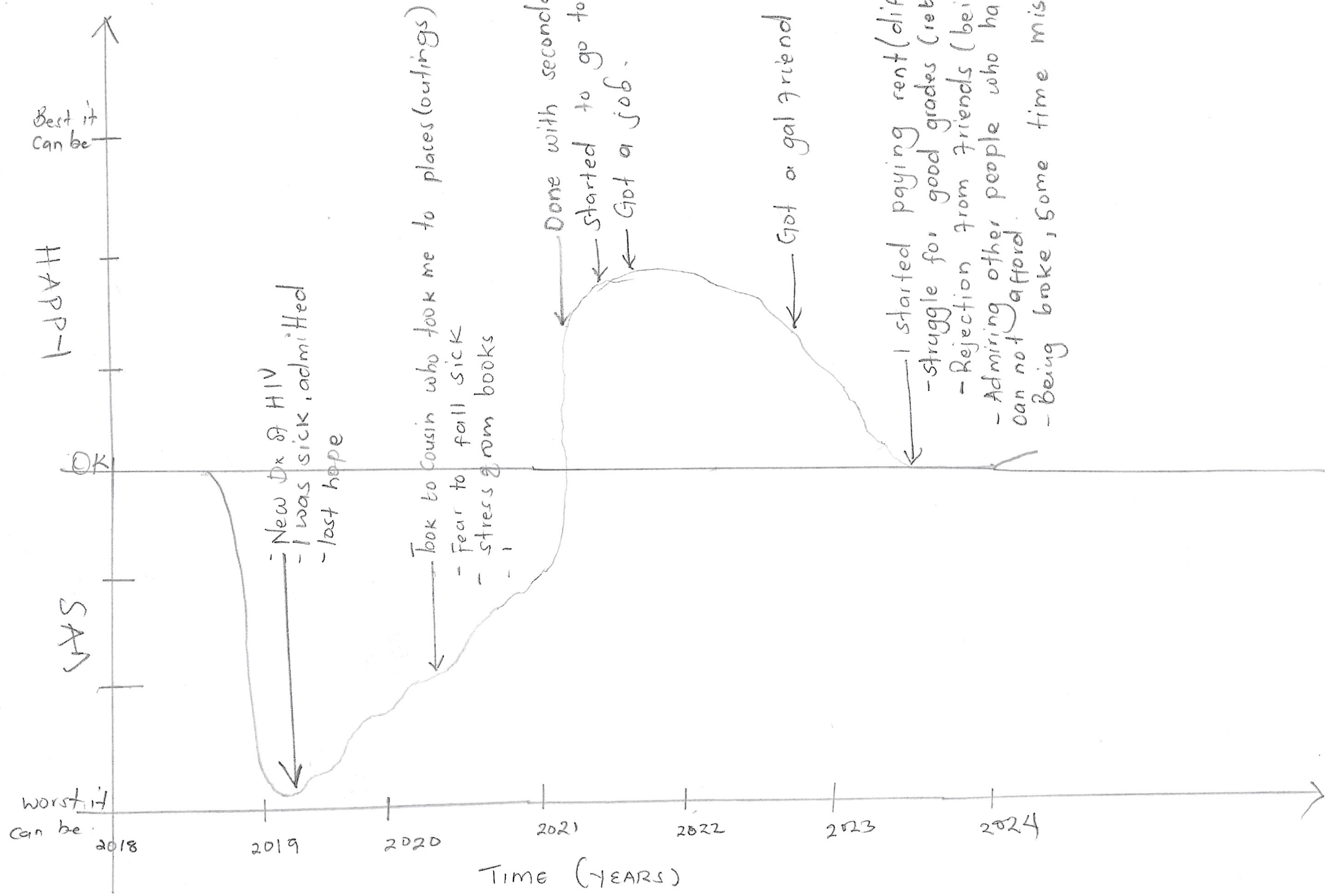

PARTICIPANT 6

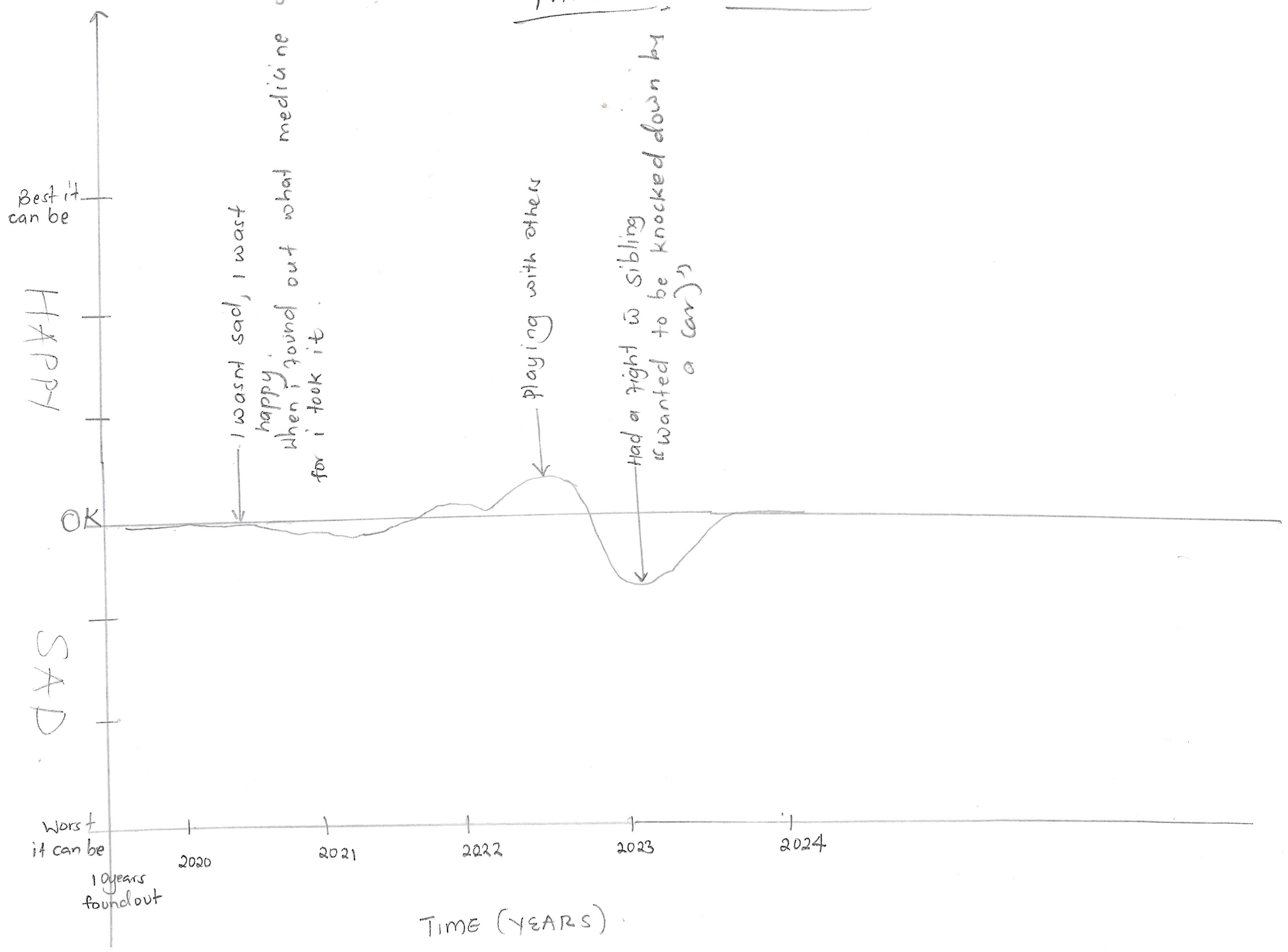

PARTICIPANT 7

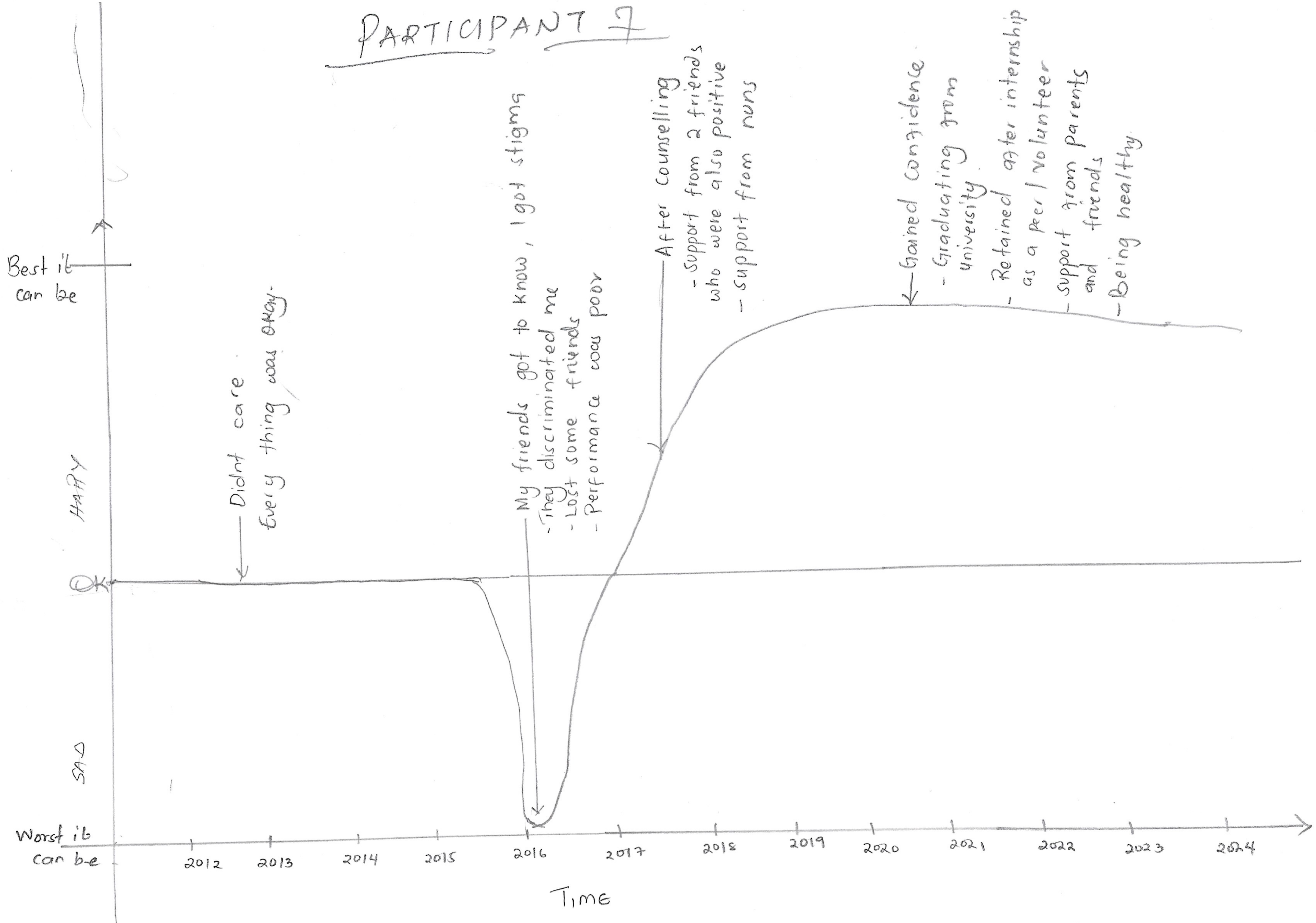

PARTICIPANT 8

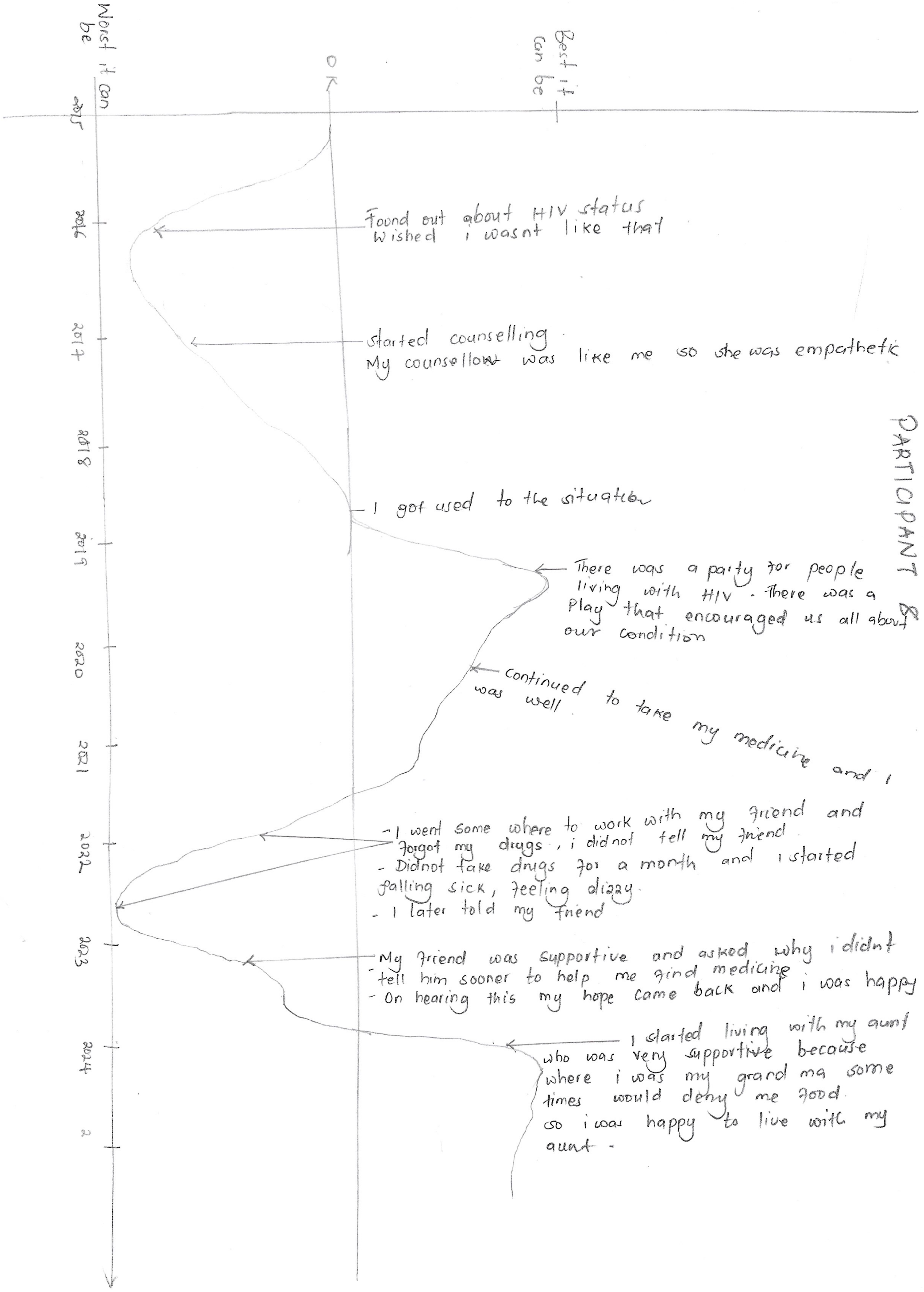

PARTICIPANT 9

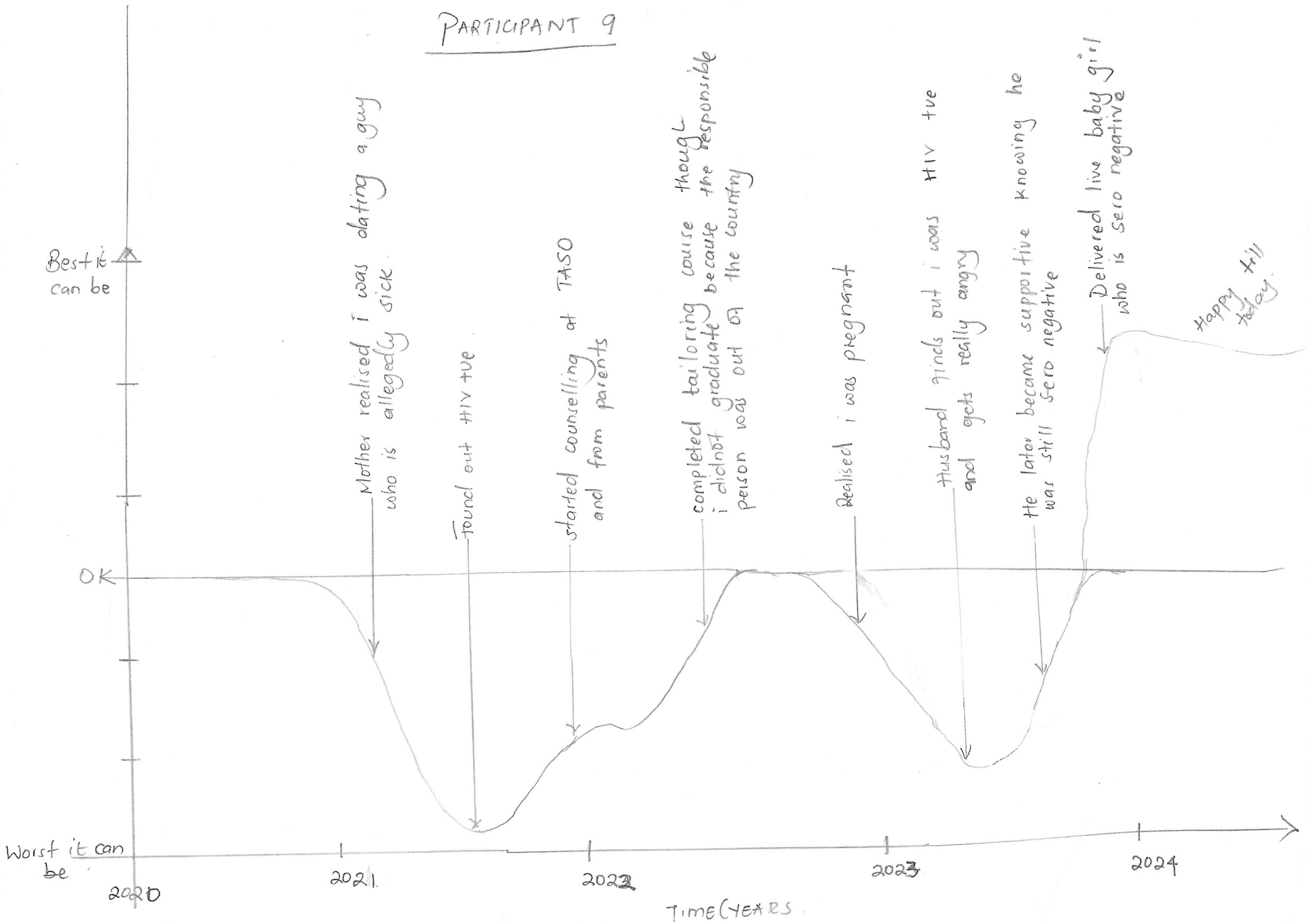

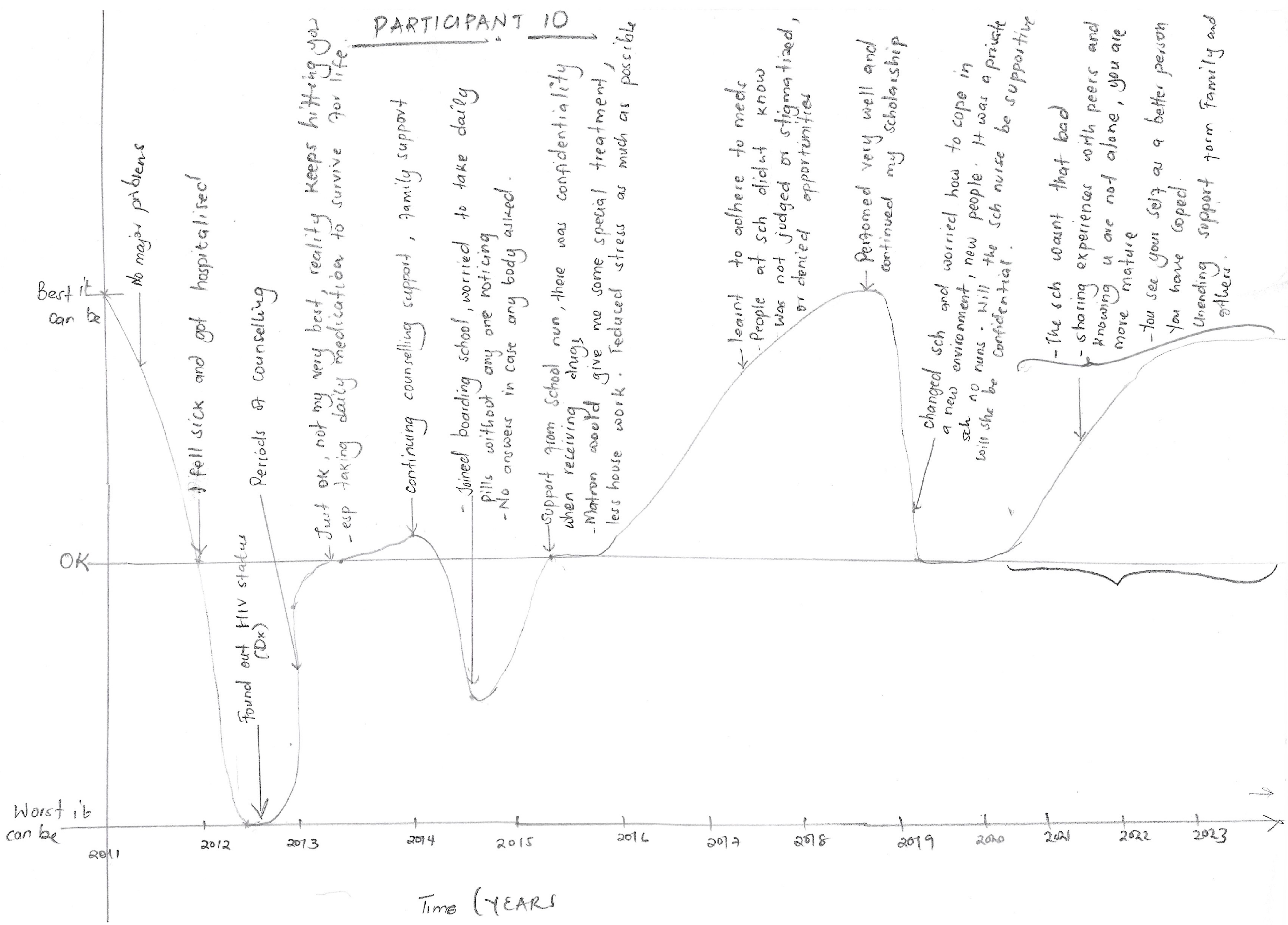
PARTICIPANT 10
Best it can be
OK
Worst it can be
Time (YEARS
2011
2012
2013
2014
2015
2016
2017
2018
2019
2020
2021
2022
2023
No major problems
I fell sick and got hospitalised
Found out HIV status (Dx)
Periods of counselling
Just ok, not my very best, reality keeps hitting you
- esp taking daily medication to survive for life.
continuing counselling support, family support
- Joined boarding school, worried to take daily pills without any one noticing
- No answers in case any body asked.
Support from school nun, there was confidentiality when receiving drugs
- Matron would give me some special treatment, less house work. reduced stress as much as possible
learnt to adhere to meds
- People at sch didn't know
- Was not judged or stigmatized, or denied opportunities
Perfomed very well and continued my scholarship
changed sch and worried how to cope in a new environment, new people. It was a private sch no nuns. Will the sch nurse be supportive will she be confidential.
- The sch wasn't that bad
- sharing experiences with peers and knowing u are not alone, you are more mature
- You see your self as a better person You have coped
Unending support form family and others.

PARTICIPANT 11

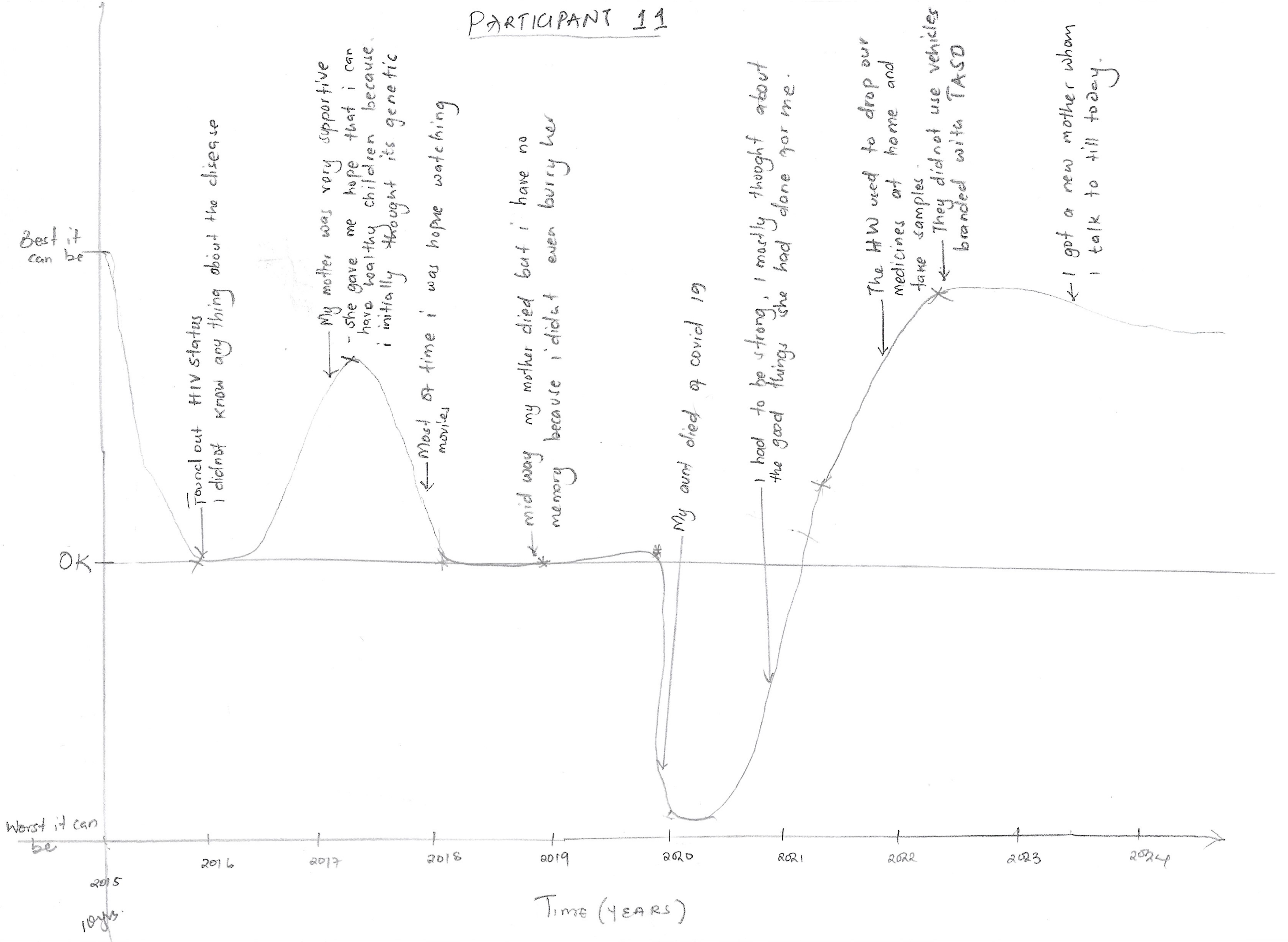

PARTICIPANT 12

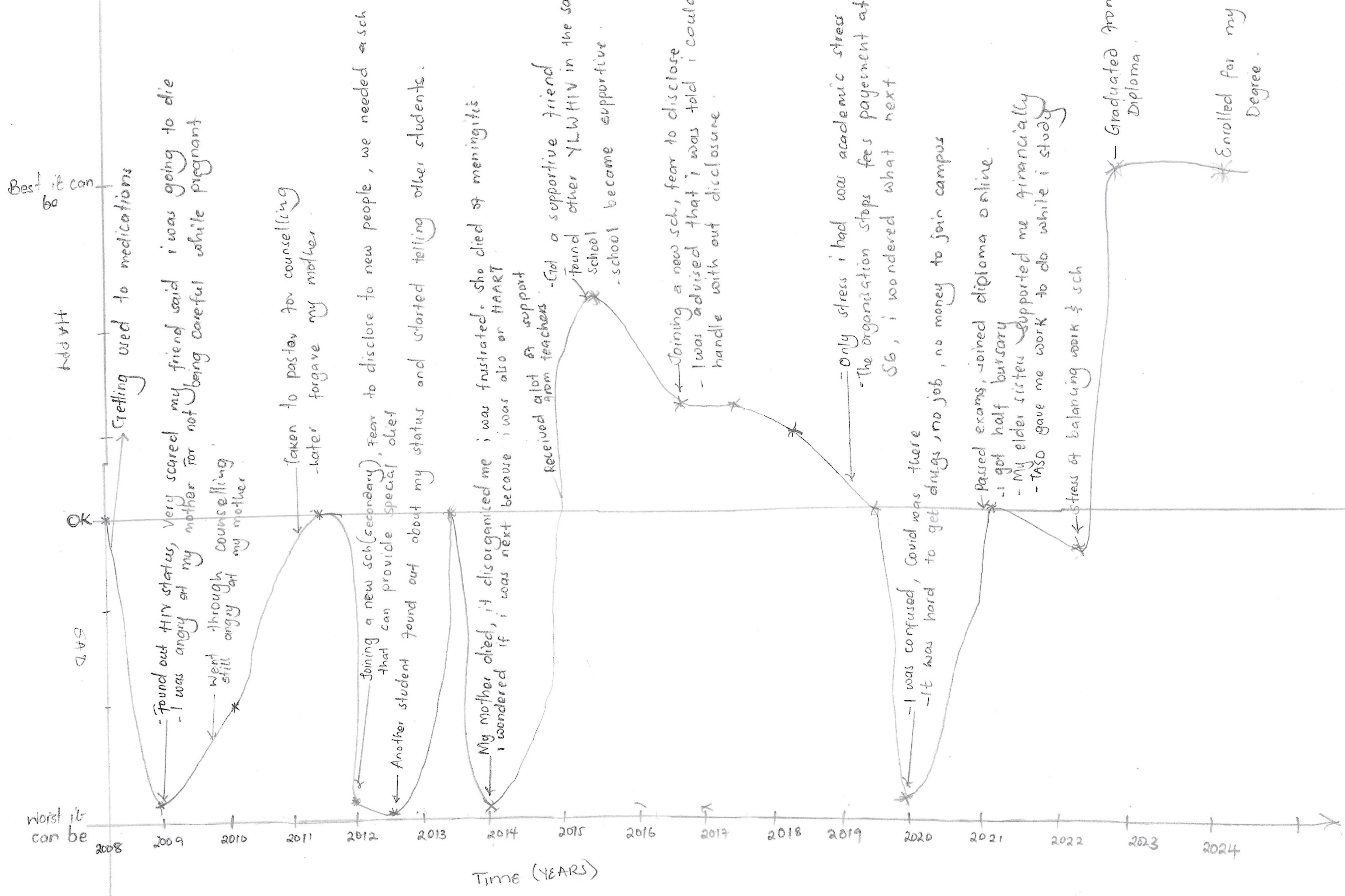

PARTICIPANT 13

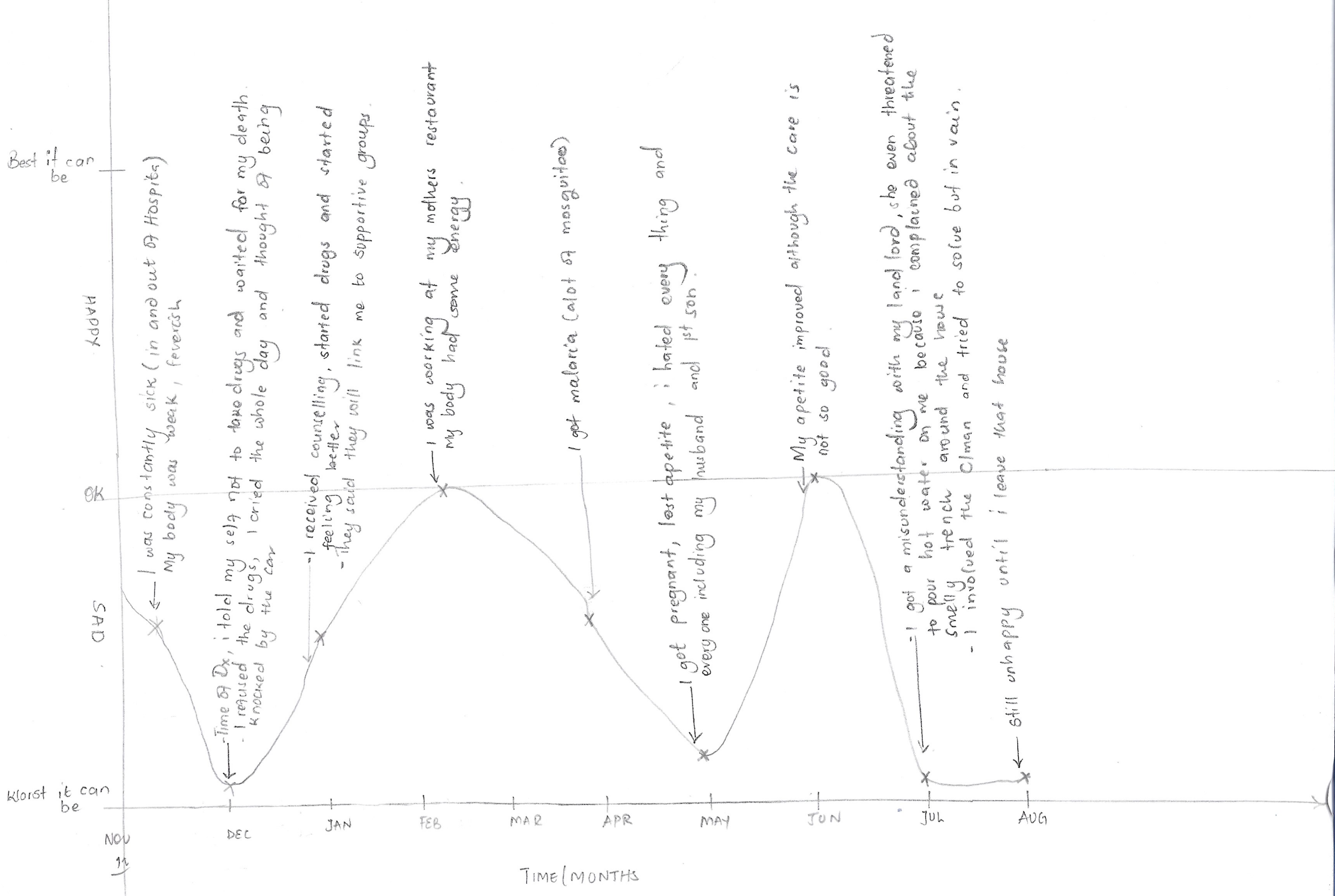

PARTICIPANT 14

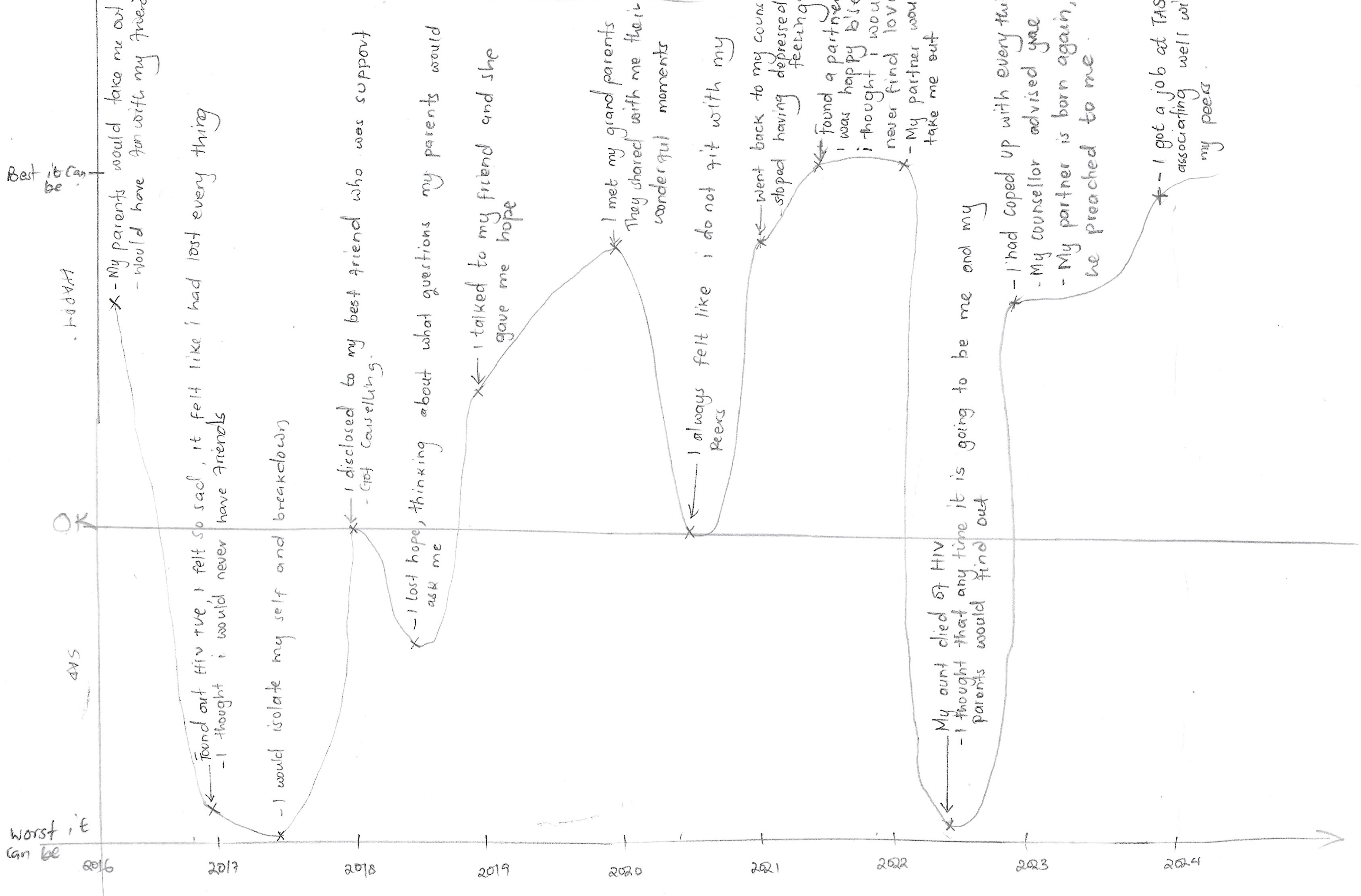

PARTICIPANT 15

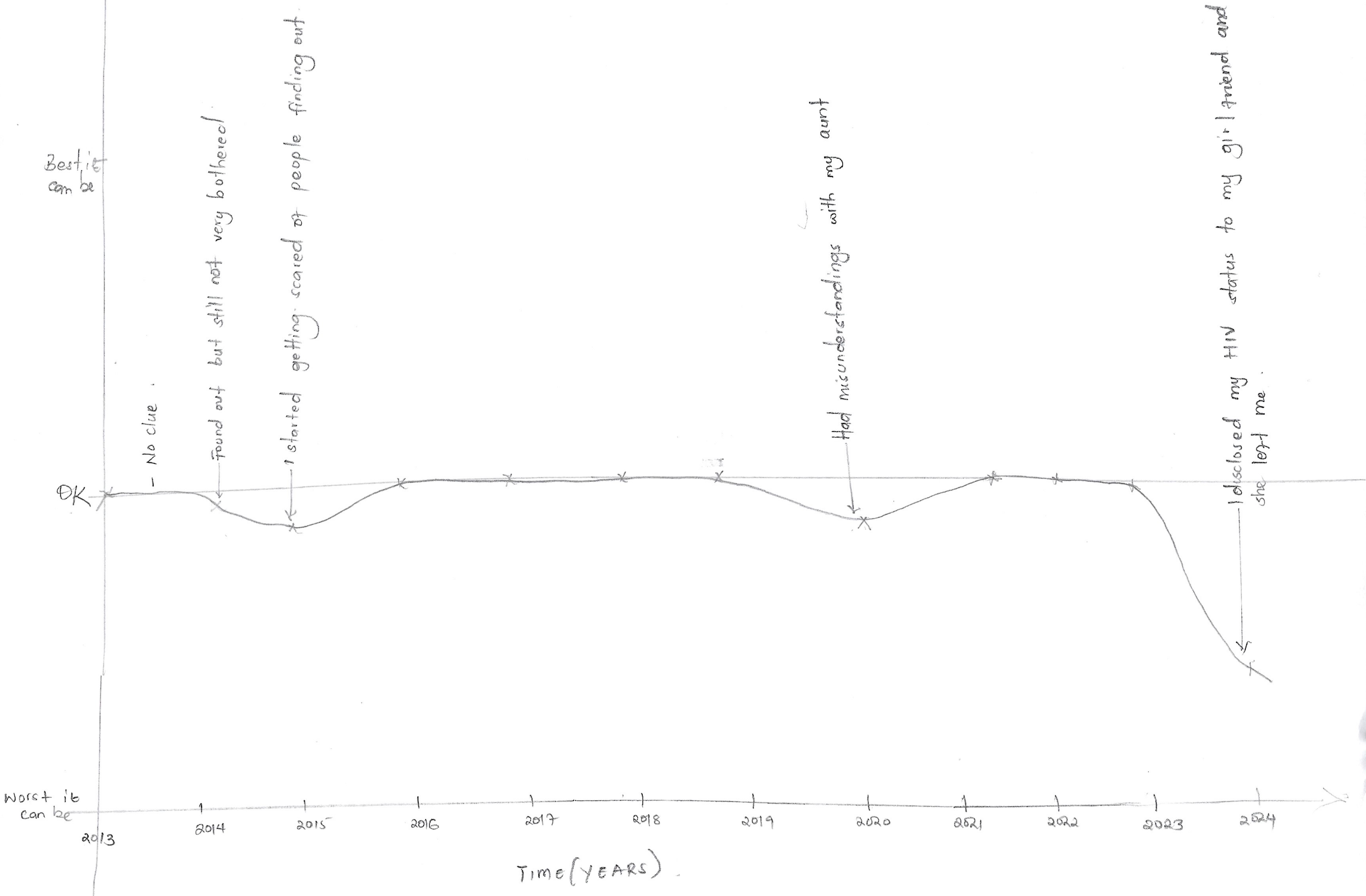

Supplement: S1 File — (PDF) [file pone.0345499.s001.pdf]
