# Supplementary material for: Crests, troughs, and plateaus: Using story theory to explore the experiences of adolescents and young people living with HIV in Kampala, Uganda
Source: PLoS One. 2026 Mar 25;21(3):e0345499. doi: 10.1371/journal.pone.0345499 (PMC13016292; doi:10.1371/journal.pone.0345499)
Supplement: Attachment — Submitted filename: PONE-D-25-36890_reviewer (1) november 2025.pdf [file pone.0345499.s002.pdf]

PLOS ONE

Crests, troughs, and plateaus: Using story theory to explore the experiences of adolescents and young people living with HIV in Kampala, Uganda

--Manuscript Draft--

Manuscript Number:	PONE-D-25-36890
Article Type:	Research Article
Full Title:	Crests, troughs, and plateaus: Using story theory to explore the experiences of adolescents and young people living with HIV in Kampala, Uganda
Short Title:	Experiences of adolescents and young people living with HIV
Corresponding Author:	Charles Peter Osingada, PhD<br>University of Minnesota Twin Cities School of Nursing<br>Blaine, Minnesota UNITED STATES OF AMERICA
Keywords:	Adolescents; Young people; Experiences; HIV; Story theory; Uganda
Abstract:	Introduction<br><br>Adolescents and young people living with HIV experience significant challenges, including unmet psychosocial and self-management needs and limited access to adequate HIV information. Peer support strategies that allow individuals to share their personal experiences and life stories have shown promise in improving their engagement in care and addressing the psychological and social challenges of living with HIV. However, current care approaches do not optimize individual life stories as a foundation for delivering patient-centered and individualized care.<br><br>Methods<br><br>This study applied a story path approach, an innovative way of exploring the experiences of adolescents and young people living with HIV. Specifically, it aimed to identify the high points, low points, and turning points in the health journeys of adolescents and young people living with HIV. This descriptive qualitative study was conducted in 2024. Data were collected through face-to-face individual interviews with adolescents and young people living with HIV and analyzed manually.<br><br>Results<br><br>Fifteen participants, aged 15 to 24 years (average age 21.4, SD 2.4), were interviewed. The findings revealed that participants experienced uplifting from social support provided by friends, family, school authorities, and peers. Despite this support, many participants experienced emotional distress, faced challenges with medication adherence, and struggled with suicidal ideation. Counseling was a vital turning point in helping nearly all participants improve their health trajectories.<br><br>Conclusions<br><br>Story theory provides a valuable framework for understanding the health experiences of adolescents and young people living with HIV and for delivering care that is tailored to their unique individual narratives. These findings may be useful to nurses, counselors, and peer supporters involved in the care of this population. Future research should explore ways to integrate story theory into adolescent HIV care services to improve health outcomes for adolescents and young people living with HIV.
Order of Authors:	Derrick Nuwahereza, BSN
	Allen Nabisere, BSN, MS
	Imelda Namatovu
	Tom Denis Ngabirano
	Charles Peter Osingada, PhD

Powered by Editorial Manager® and ProduXion Manager® from Aries Systems Corporation

Additional Information:	
**Question**	**Response**
**Financial Disclosure**<br><br>Enter a financial disclosure statement that describes the sources of funding for the work included in this submission. Review the submission guidelines for detailed requirements. View published research articles from *PLOS ONE* for specific examples.<br><br>This statement is required for submission and **will appear in the published article** if the submission is accepted. Please make sure it is accurate.<br><br>**Funded studies**<br>Enter a statement with the following details:<br>• Initials of the authors who received each award<br>• Grant numbers awarded to each author<br>• The full name of each funder<br>• URL of each funder website<br>• Did the sponsors or funders play any role in the study design, data collection and analysis, decision to publish, or preparation of the manuscript?<br><br>Did you receive funding for this work?	The author(s) received no specific funding for this work.
**Competing Interests**<br><br>Use the instructions below to enter a competing interest statement for this submission. On behalf of all authors, disclose any competing interests that could be perceived to bias this work—acknowledging all financial support and any other relevant financial or non-financial competing interests.<br><br>This statement is required for submission and **will appear in the published article** if the submission is accepted. Please make sure it is accurate and that any funding sources listed in your Funding Information	The authors have declared that no competing interests exist.

later in the submission form are also declared in your Financial Disclosure statement.<br><br>View published research articles from *PLOS ONE* for specific examples.<br><br>**NO authors have competing interests**<br><br>Enter: *The authors have declared that no competing interests exist.*<br><br>**Authors with competing interests**<br><br>Enter competing interest details beginning with this statement:<br><br>*I have read the journal's policy and the authors of this manuscript have the following competing interests: [insert competing interests here]*<br><br>* typeset	
**Ethics Statement**<br><br>Enter an ethics statement for this submission. This statement is required if the study involved:<br><br>• Human participants<br>• Human specimens or tissue<br>• Vertebrate animals or cephalopods<br>• Vertebrate embryos or tissues<br>• Field research<br><br>Write "N/A" if the submission does not require an ethics statement.<br><br>General guidance is provided below. Consult the submission guidelines for detailed instructions. **Make sure that all information entered here is included in the Methods section of the manuscript.**	Ethics approval was obtained from the Makerere University School of Health Sciences Research Ethics Committee (MAKSHSREC-2024-679), and administrative clearance was obtained from the management of TASO Mulago branch. In addition, written informed consent was obtained from the participants before they participated in the study. Furthermore, assent was sought from participants who were less than 18 years old, and their parents or guardians were requested to provide permission for their child to be involved in the study.

Powered by Editorial Manager® and ProduXion Manager® from Aries Systems Corporation

**Format for specific study types**<br><br>**Human Subject Research (involving human participants and/or tissue)**<br>• Give the name of the institutional review board or ethics committee that approved the study<br>• Include the approval number and/or a statement indicating approval of this research<br>• Indicate the form of consent obtained (written/oral) or the reason that consent was not obtained (e.g. the data were analyzed anonymously)<br><br>**Animal Research (involving vertebrate animals, embryos or tissues)**<br>• Provide the name of the Institutional Animal Care and Use Committee (IACUC) or other relevant ethics board that reviewed the study protocol, and indicate whether they approved this research or granted a formal waiver of ethical approval<br>• Include an approval number if one was obtained<br>• If the study involved *non-human primates*, add *additional details* about animal welfare and steps taken to ameliorate suffering<br>• If anesthesia, euthanasia, or any kind of animal sacrifice is part of the study, include briefly which substances and/or methods were applied<br><br>**Field Research**<br><br>Include the following details if this study involves the collection of plant, animal, or other materials from a natural setting:<br>• Field permit number<br>• Name of the institution or relevant body that granted permission	
**Data Availability**<br><br>Authors are required to make all data underlying the findings described fully available, without restriction, and from the time of publication. PLOS allows rare exceptions to address legal and ethical concerns. See the PLOS Data Policy and FAQ for detailed information.	Yes - all data are fully available without restriction

A Data Availability Statement describing where the data can be found is required at submission. Your answers to this question constitute the Data Availability Statement and **will be published in the article**, if accepted.<br><br>**Important:** Stating 'data available on request from the author' is not sufficient. If your data are only available upon request, select 'No' for the first question and explain your exceptional situation in the text box.<br><br>Do the authors confirm that all data underlying the findings described in their manuscript are fully available without restriction?	
**Describe where the data may be found in full sentences. If you are copying our sample text, replace any instances of XXX with the appropriate details.**<br><br>• If the data are **held or will be held in a public repository**, include URLs, accession numbers or DOIs. If this information will only be available after acceptance, indicate this by ticking the box below. For example: *All XXX files are available from the XXX database (accession number(s) XXX, XXX.).*<br>• If the data are all contained **within the manuscript and/or Supporting Information files**, enter the following: *All relevant data are within the manuscript and its Supporting Information files.*<br>• If neither of these applies but you are able to provide **details of access elsewhere**, with or without limitations, please do so. For example:<br><br>*Data cannot be shared publicly because of [XXX]. Data are available from the XXX Institutional Data Access / Ethics Committee (contact via XXX) for researchers who meet the criteria for access to confidential data.*<br><br>*The data underlying the results presented in the study are available from (include the name of the third party*	All relevant data are within the manuscript and its Supporting Information files

and contact information or URL).<br>• This text is appropriate if the data are owned by a third party and authors do not have permission to share the data.<br><br>* typeset	
Additional data availability information:	

Powered by Editorial Manager® and ProduXion Manager® from Aries Systems Corporation

Edited manuscript Click here to access/download;Manuscript;Manuscript document.docx

Crests, troughs, and plateaus: Using story theory to explore the experiences of adolescents and young people living with HIV in Kampala, Uganda

Derrick Nuwahereza[1], Allen Nabisere[1], Imelda Namatovu[1], Tom Denis Ngabirano[1], Charles Peter Osingada[2*]

Author affiliations

[1] Department of Nursing, School of Health Sciences, Makerere University College of Health Sciences, Kampala, Uganda.

[2] School of Nursing, University of Minnesota,

Weaver Densford Hall, 308 Harvard St SE, Minneapolis, Minnesota, USA.

*Correspondence author

Email: Osing004@umn.edu

Authorship contributions

D. N. conceptualized the study, collected, and participated in data analysis; A.N supported data collection and analysis, reviewed and edited the manuscript; I.N collected data and participated in data analysis; T.D.N reviewed the draft manuscript. C.P.O conceptualized the study, supported during data collection and analysis, and drafted and reviewed the manuscript. All authors reviewed the final version of the manuscript.

Abstract

Introduction: Adolescents and young people living with HIV experience significant challenges, including unmet psychosocial and self-management needs and limited access to adequate HIV information. Peer support strategies that allow individuals to share their personal experiences and life stories have shown promise in improving their engagement in care and addressing the psychological and social challenges of living with HIV. However, current care approaches do not optimize individual life stories as a foundation for delivering patient-centered and individualized care.

Methods: This study applied a story path approach, an innovative way of exploring the experiences of adolescents and young people living with HIV. Specifically, it aimed to identify the high points, low points, and turning points in the health journeys of adolescents and young people living with HIV. This descriptive qualitative study was conducted in 2024. Data were collected through face-to-face individual interviews with adolescents and young people living with HIV and analyzed manually.

Results: Fifteen participants, aged 15 to 24 years (average age 21.4, SD 2.4), were interviewed. The findings revealed that participants experienced uplifting from social support provided by friends, family, school authorities, and peers. Despite this support, many participants experienced emotional distress, faced challenges with medication adherence, and struggled with suicidal ideation. Counseling was a vital turning point in helping nearly all participants improve their health trajectories.

Conclusions: Story theory provides a valuable framework for understanding the health experiences of adolescents and young people living with HIV and for delivering care that is tailored to their unique individual narratives. These findings may be useful to nurses, counselors, and peer supporters involved in the care of this population. Future research should explore ways to integrate story theory into adolescent HIV care services to improve health outcomes for adolescents and young people living with HIV.

Keywords: Adolescent, Young people, Experiences, HIV, Story theory, Uganda

Introduction

By the end of 2023, approximately 40 million people were living with HIV globally, and another 630,000 individuals had died of AIDS-related illnesses [1]. East and Southern Africa continue to bear the highest burden of the disease, with approximately 20.8 million people living with HIV [1]. Although Sub-Saharan Africa has registered the biggest overall decline in new HIV infections and AIDS-related deaths [2], adolescent girls and young women in East and Southern Africa remain disproportionately affected by the disease [1]. Besides having a high incidence of HIV, adolescents and young people living with HIV (AYPLHIV) face significant challenges, including unmet psychosocial and self-management needs, as well as limited access to adequate information about HIV.

In Uganda, by the end of 2022, approximately 1.4 million people were living with HIV, including 166,000 adolescents and young people aged 15-24 years [3]. Although nationally there has been a significant decline in new infections and AIDS-related deaths, the incidence of HIV among young Ugandans remains alarmingly high. The government of Uganda prioritizes innovative approaches to reach AYPLHIV with comprehensive prevention, care, and treatment services [4]. Currently in most HIV care and treatment facilities in Uganda, the Young People and Adolescent Peer Supporters (YAPS) model [4] is being implemented. Adapted from Zimbabwe's Community Adolescent Treatment Supporters (CATS) trial [5], the YAPS model leverages young people and adolescents as key service providers to screen, test, and link their peers to care. According to a recent Uganda Aids Commission (UAC) report, the model has resulted in increased case identification, linkage to care, retention in treatment, and improved viral load suppression among AYPLHIV [4].

The success of the YAPS model and similar peer support programs [6] is partly due to the ability of peer supporters to share their experiences and life stories, positioning themselves as credible sources of information and role models for AYPLHIV[7]. The impact of the YAPS model can be enhanced by weaving intentional approaches that encourage peer supporters and healthcare providers to actively engage in dialogue with their patients to understand their personal narratives. When peer supporters and healthcare providers listen to the stories of young people living with HIV, they foster a deeper understanding of the challenges they encounter and facilitate individualized care. This patient-centered approach helps young individuals feel seen, valued, and more willing to engage during medical visits. Additionally, the understanding that develops allows providers to generate care plans that address both the clinical and psychosocial needs of AYPLHIV, thereby improving their physical and mental health.

To date there has been insufficient exploration of the potential within the personal narratives of young people living with HIV to shape patient-centered care. The lack of understanding of individual patient's story hampers the development of targeted interventions and support systems. Personal stories reveal the intricate pathways individuals navigate, shedding light on their unique encounters with stigma, and struggles with mental health and social relationships. Training young people and adolescent peer supporters to elicit and engage with these personal stories helps them to gain deeper insights into the psychosocial challenges faced by AYPLHIV. This enables them to deliver more personalized, and efficacious interventions aimed at enhancing the overall well-being of AYPLHIV.

The objective of this study was to applying story theory [8] to explore the experiences of adolescents and young people living with HIV. Specifically, the study focused on identifying the high points, low points, and turning points in the journeys of AYPLHIV. By collecting and

analyzing their story paths and narratives, the investigators aimed to understand the complexities of the experiences of AYPLHIV and gain a comprehensive understanding of the challenges they face. Insights reported in this study may help nurses, peer supporters, and other health providers in designing targeted interventions and providing anticipatory counseling tailored to the unique circumstances of these young individuals. Additionally, findings from this study may function to elevate the utility of story theory in the care of AYPLHIV.

Materials and Methods

Study design and setting

This was a descriptive qualitative study. This design was suitable as it allowed investigators to present straight descriptions of the experiences of participants and staying close to the data without engaging in deep interpretations [9]. This study was conducted at the AIDs Support Organization (TASO) Centre, located in Mulago. The center provides services for patients living within a 75 km radius reach and its catchment area covers the districts of Kampala, Mityana, Mukono, and Wakiso. TASO Mulago provides HIV/AIDS clinical and psychological services to children, adolescents, youths, and adults living with HIV/AIDS. Examples of the services include adherence counseling, condom education and distribution, health education, adherence measurement, tuberculosis screening, opportunistic infections examinations, and laboratory investigations. The clinic days for AYPLHIV are Tuesday, Wednesday, and Thursday. On clinic days, about 60-80 patients come for ART refills and other HIV/AIDS services.

Study participants

The study was conducted among AYPLHIV aged 15-24 years, that were receiving care and treatment services from TASO. To be included in the study, prospective participants had to

be aged 15-24 years, have lived with HIV for at least six months, able to speak English or Luganda, receiving care and treatment from the TASO-Uganda Mulago branch, able to provide informed consent or assent, and willing to participate. Patients who were unable to communicate verbally were excluded. Participants were purposefully identified from those attending adolescent-friendly care services in the study site. Participants were identified with the help of health personnel in the clinics.

Data collection

Data collection took place from 30th July to 7th August 2024. In-depth face-to-face interviews were conducted with consenting participants at the health facility by N.I. The interviews were conducted in a room at the study site that offered sufficient auditory privacy. The interview guide used during the interview was developed using guidance provided by the developers of the story theory [8]. Participants were invited to reflect on their present and past experiences, describe their future hopes and dreams. Each interview was audio recorded after obtaining permission from the participant. The interview time ranged from 15 to 44 minutes. Additionally, each participant was requested to recall their experiences from the time they got to know their HIV positive status. Each participant was requested to map these experiences on a plain piece of paper provided by the interviewer. Specifically, they were instructed to mark the high points - times when things were going well, low points- when things were not going well, and turning points - deciding moments or shifts in perspective that altered life direction [10]. Before ending the interview, the interviewer looked over the story path created by each participant to ensure accuracy of what had been written. After the interview, each participant was paid 5,000 Uganda shillings (about 1.3 US dollars) to compensate for their time. At the beginning of the study, the investigators had planned to recruit 15 to 20 participants. However,

after conducting 15 interviews, data collection was stopped because no new experiences were emerging from the interviews, indicating that a point of data saturation had been attained [11]. This paper reports findings from analysis of story paths drawn by the participants.

Data management and analysis

The audio recordings were transcribed by N.I. and N.A., resulting in 15 complete transcripts. Each transcript was cross-checked against the original recording to ensure accuracy and completeness. To gain an overall understanding of the data, each transcript was read in its entirety. OCP then applied a deductive coding approach to manually identify the high points, low points, and turning points in the health trajectories of adolescents and young people living with HIV (AYPLHIV). During coding, these key points were highlighted using different colors to distinguish them. Once all transcripts were coded, the codes were grouped into categories based on whether they represented high, low, or turning points. In addition, each individual story path was manually analyzed to further identify these points. A summary of the findings, including the identified high, low, and turning points, is presented in the results section, accompanied by illustrative quotes.

Ethics approval

Ethics approval was obtained from the Makerere University School of Health Sciences Research Ethics Committee (MAKSHSREC-2024-679), and administrative clearance was obtained from the management of TASO Mulago branch. In addition, written informed consent was obtained from the participants before they participated in the study. Furthermore, assent was sought from participants who were less than 18 years old, and their parents or guardians were requested to provide permission for their child to be involved in the study.

Results

Fifteen participants were interviewed, with ages ranging from 15 to 24 years. The average age was 21.4 years, with a standard deviation of 2.4 years. Nearly three-quarters of the participants were single females. Ten participants (67%) had been living with HIV since birth. Additional sociodemographic characteristics are presented in Table 1.

Table 1: Demographic characteristics of study participants

Participant Number	Age	Marital status	Sex	Current level of education	Duration lived with HIV
1	22	Single	Female	Secondary	13 months
2	21	Married	Female	Secondary	Since birth
3	23	Single	Female	Primary	Since birth
4	24	Single	Female	University	Since birth
5	21	Single	Male	University	60 months
6	15	Single	Female	Primary	Since birth
7	23	Single	Female	University	Since birth
8	19	Single	Male	Secondary	Since birth
9	22	Married	Female	Secondary	36 months
10	22	Single	Female	University	Since birth
11	18	Single	Male	Secondary	Since birth
12	24	Single	Female	University	Since birth
13	22	Married	Female	Primary	9 months
14	21	Married	Female	University	84 months
15	24	Single	Male	University	Since birth

High points

For most of the participants, moments of social and emotional support played a crucial role, with participants frequently mentioning the encouragement and care received from family members, health providers, school personnel, and friends as uplifting.

"We had a doctor here she was called... she also helped me in times when I felt like why am I taking this medicine, she used to call me every day she used to follow me up, in fact she used to call me for those first 2 weeks she used to call me every day to ask how you taken the medicine? how are you feeling now? She even used to give some ka money. For the first weeks

when I came back, she told me now you see you are fine the viral load is going low, she told me next time”. ***(Participant 5; Male, 21 years)***

“…when I was well after the sickness, they brought me here I started taking drugs then they took me to my brothers place …to gain confidence, he used to counsel me, …he used to take me to places like forest park those interesting places and he told me everything is going to be okay nothing is going to change apart from just taking drugs… those two months made me feel confident I realized that everything is okay I will be fine then I let go, I had to accept the situation”. ***(Participant 5; Male, 21 years)***

“...she always advised me and whenever she's seeing that... people discussed about HIV things and then maybe sometimes the discussion will just come as one of the disease people are learning in biology. But you know, you feel as if they are talking about you, this kind of thing. She would know that it is touching me. Sometimes she comes after the lesson and says, are you okay? Are you fine? She would want to find out how you are. Yeah. And then also during time for drugs… she's the one who was now reminding me. If we are in prep time and we are still revising, and she has seen it's 9 o'clock. She will tap me. She'll come and say, can we go? She will not tell anyone where we are going. She just holds my hand and moves out, as if we are going maybe for a short call or to discuss something. Yet she's reminding me, she wants me to go and take my drugs. Then she comes back and sits in the class”. ***(Participant 12, Female, 24 years)***

In addition, securing a source of income, disclosing HIV status, and receiving positive reinforcement from loved ones also emerged as meaningful high points in the experiences of adolescent and young people living with HIV.

“…having friends who encourage you to continue doing what you are doing because i have a friend who is older, we met in school, he stays in...(name of location omitted) when we talk, he tells me to continue taking my medicine. He gives me hope”. ***(Participant 8; Male, 19 years)***

Low points

Most of participants experienced low moments upon discovering their HIV-positive status. Many struggled with adherence to medication due to stigma, forgetfulness, or the challenges posed by boarding school environments. Experiences of rejection, whether from friends, romantic partners, or the workplace, were also commonly reported, illustrating the pervasive impact of stigma and discrimination.

*"The challenges I have faced there is a lady I used to work with, she kept telling my customers that am HIV positive I felt really bad because they started discriminating me, disrespecting me but I left that place where I did not feel welcomed that is the only challenge I have faced". **(Participant 3, Female, 23 years)***

*"Automatically bad, I became emotional, I stigmatized myself, I hated everyone around me, that's how it was". **(Participant 4, Female, 24 years)***

Additionally, several participants expressed suicidal ideation, feelings of hopelessness, and self-isolation, underscoring the severe psychological toll associated with HIV diagnosis. Falling sick with illnesses related or unrelated to HIV was also a common low point for this population of adolescents as it increased their fears and worries of losing their lives.

*"I actually felt like committing suicide, it was too much for me and the person that gave it to me actually knew that was the worst thing for me to bear but I went through therapy. I had like 3months of therapy so I just had to accept my condition and move on…. I was broken and I was like did this person not know he was sick. So, I don't know (crying). I went through therapy, I was so depressed I couldn't open up to anyone, couldn't open up to my mum so i told a friend and a friend convinced me to at least tell my mother". **(Participant 1, Female, 22 years)***

"…of course (sighs) hmmn hmmn, I felt like it was over for me, I felt like the world had ended, eeh and I felt like even killing myself it was really a bad situation cause I was totally sick at that time the body was so soft, it had made me go down so I felt like I won't even get well even

*if I take tablets but later on I was counselled and got some help". **(Participant 5; Male, 21 years)***

Turning points

The turning points reported by participants predominantly revolve around key moments of social support, counseling, and personal acceptance of their HIV-positive status. Many participants identified receiving counseling as a pivotal moment in their lives, helping them cope with their diagnosis, adhere to medication, and manage emotional distress.

*"…I was feeling bad they discriminated me , I was not feeling okay even I wanted to change the school yea but later on our sister those nuns they were like no you do this they counselled me and the nurse was there for me and some of my friends because there were some 2 girls who were also swallowing [taking antiretroviral medicines], they were there for me and I picked up. It wasn't easy….the bigger part was counselling because I used to go to the dormitory and cry after crying, I sleep yea like that. Even if you tried to concentrate and read it wasn't easy. So I used to cry sleep, cry sleep" **(Participant 7; Female, 23 years)***

*"When the counselor spoke to me and gave me hope that I can still live with it and told me, you just continue with your medication, like do not abandon yourself from people, you continue speaking to them because there is nothing, like if you take your medication, nothing really happens and maybe if... anything has gone wrong, come back and speak to me. If you cannot tell your friend, come back and open up to me" **(Participant 14; Female, 21 years)***

Acceptance of HIV status and disclosure to supportive individuals, including family members, friends, and peers living with HIV, also played a crucial role in fostering resilience and instilling hope.

"I accepted it, acceptance was the major thing for me and when I interact with some of my friends that I met here that are HIV positive, it gave me a relief because they have this thing of its just, just take your medication and move on just take the tablet and the rest is okay and the fact that I don't have any other health problem apart from it yea. So, they made it simpler for me,

taking my medication and accepting to live with it, only the two things that I have to follow" ***(Participant 1, Female, 22 years)***

"What matters most, the first thing was acceptance that one gave me hard time but…it came after some good time because even I finished my candidate classes when am in day school when I had not felt like I can keep my own medicine at school. I felt like aah aah acceptance came when I was in A level that's when I accepted, I was like aahh now come what may am not going backwards it has come and it will never go". ***(Participant 5; Male, 21 years)***

Discussion

This study utilized story theory to explore the experiences of adolescents and young people living with HIV (AYPLHIV). Specifically, we sought to document the high, low, and turning points in their health trajectories. Our findings revealed that the most frequently mentioned high points were receiving encouragement and care from family members, school personnel, and peer networks. Disclosure of HIV status and receiving positive reinforcement from loved ones also emerged as a commonly cited high point. Conversely, most participants experienced their lowest moments upon discovering their HIV-positive status. Additional low points included struggles with medication adherence due to stigma, forgetfulness, and the challenges posed by boarding school environments. Experiences of rejection from friends, romantic partners, and workplaces were also reported. Moreover, several participants expressed suicidal ideation, feelings of hopelessness, and self-isolation, underscoring the severe psychological toll associated with an HIV diagnosis.

Turning points in participants' health trajectories predominantly revolved around key moments of social support, counseling, and personal acceptance of their HIV-positive status. Many participants identified receiving counseling as a pivotal moment, helping them cope with their diagnosis, adhere to medication, and manage emotional distress. Disclosure to supportive

individuals, including family members, friends, and peers living with HIV, also played a crucial role in shifting the trajectory of their health journeys.

Our findings indicate that most participants experienced uplifting moments when they received social support from friends, family members, school personnel, and peers. This aligns with existing literature on the importance of social and peer support for AYPLHIV. For example, Willis et al. (2019) found that adolescents who received support from community adolescent treatment supporters experienced improved psychosocial well-being compared to those who did not receive such support [12]. Additionally, social and peer support have been linked to better medication adherence [13], improved adherence efficacy [14], viral load suppression [15, 16] and reduced depression [17]. Path stories analyzed in this study indicated that almost all participants experienced a trough period soon after learning their HIV status. This highlights the importance of linking adolescents and young people to social support services immediately following diagnosis. Early linkage to these services may mitigate the magnitude of psychosocial distress and enhance the potential for a quicker recovery.

Our study also revealed that AYPLHIV frequently experienced low points characterized by nonadherence to medication due to fear of stigma, as well as high levels of suicidal ideation, depression, and hopelessness. These findings are consistent with previous reports documenting the prevalence and spectrum of mental health challenges faced by AYPLHIV[18–21]. While multiple studies have established the mental health burden among this population, few have provided insight into the specific timing of these challenges. By using graphical approaches such as story paths, healthcare providers may be better equipped to offer anticipatory care tailored to the evolving needs of AYPLHIV.

For most adolescents interviewed, receiving counseling was a pivotal turning point, facilitating coping with their diagnosis, adherence to medication, and the management of emotional distress. This finding underscores the importance of HIV counseling services as a critical mechanism for promoting behavior change and psychological well-being [22, 23]. The story paths analyzed in this study depict health trajectories marked by crests, troughs, and plateaus, highlighting the need for ongoing counseling and sustained support particularly during the low periods to help AYPLHIV lead meaningful and fulfilling lives.

Our study had a few limitations that should be considered when interpreting the findings. First, we relied on participants' ability to recall their experiences from the time they first learned of their HIV-positive status. Given that some participants had been living with the disease for more than seven years, their recollection of past events may not have been entirely accurate, which could have affected the quality of the findings. However, the use of a visual mapping approach may have helped participants organize their memories more clearly, which likely improved their ability to identify key experiences they might have otherwise overlooked. Second, despite efforts to balance participant gender, males were underrepresented in our study. This limited our ability to fully explore and capture gender-specific variations in the experiences of adolescents and young people living with HIV (AYPLHIV). Finally, some participants, particularly those with only a primary level of education, struggled with drawing story paths and required support from the research assistants.

Conclusion

Story theory provides a valuable framework for understanding the health experiences of adolescents and young people living with HIV and for delivering care that is tailored to their unique individual narratives. Using this middle-range theory, our study revealed that AYPLHIV

benefit greatly from social support provided by friends, family, school authorities, and peers. At the same time, many experienced emotional distresses after learning their HIV-positive status, faced challenges with medication adherence, and, in some cases, struggled with suicidal ideation. Counseling emerged as a crucial factor in helping nearly every participant improve their health trajectory. The findings from this study highlight the potential of story theory to enhance adolescent-friendly HIV care services. Future research should explore how integrating story theory into these services could impact the health outcomes of this population.

Acknowledgments

The authors wish to acknowledge the participants of this study for their contribution of time and invaluable perspectives.

List of abbreviations

AYPLHI – Adolescents and young people Living with HIV, CATS – Community Adolescent Treatment Supporters, HIV- Human Immunodeficiency Virus, TASO- The AIDS Support Organization, UAC- Uganda AIDS Commission.

Availability of data and materials

The datasets used and/or analyzed during the current study are available from the corresponding author on reasonable request.

Conflict of Interest

The authors declare that they have no competing interests.

Funding statement: This study did not external funding

References

1. The Joint United Nations AIDS Programme (UNAIDS). The Urgency of Now: AIDS at a Crossroads. 2024.

2. Wasswa H. Sub-Saharan Africa registers biggest declines in AIDS infections, says UNAIDS. BMJ. 2023;383:p2843.

3. The Uganda Aids Commission (UAC). Facts on HIV and AIDS in Uganda. 2023.

4. The Uganda Aids Commission (UAC). Annual joint AIDS review report. 2023; November.

5. Mavhu W, Willis N, Mufuka J, Bernays S, Tshuma M, Mangenah C, et al. Effect of a differentiated service delivery model on virological failure in adolescents with HIV in Zimbabwe (Zvandiri): a cluster-randomised controlled trial. Lancet Glob Heal. 2020;8:e264–75. doi:10.1016/S2214-109X(19)30526-1.

6. Burke VM, Frimpong C, Miti S, Mwansa JK, Abrams EA, Merrill KG, et al. "It must start with me, so it started with me": A qualitative study of Project YES! youth peer mentor implementing experiences supporting adolescents and young adults living with HIV in Ndola, Zambia. PLoS One. 2022;17 2 February:1–12. doi:10.1371/journal.pone.0261948.

7. Mark D, Hrapcak S, Ameyan W, Lovich R, Ronan A, Schmitz K, et al. Peer Support for Adolescents and Young People Living with HIV in sub-Saharan Africa: Emerging Insights and a Methodological Agenda. Curr HIV/AIDS Rep. 2019;16:467–74.

8. Smith MJ, Liehr PR. Story Theory. Middle Range Theory Nursing, Fifth Ed. 2023;26506:191–209.

9. Sandelowski M. Focus on research methods: Whatever happened to qualitative description?

Res Nurs Heal. 2000;23:334–40.

10. Liehr P, Smith MJ. Claiming the Narrative Wave with Story Theory. Adv Nurs Sci. 2020;43:15–27.

11. Moser A, Korstjens I. Series: Practical guidance to qualitative research. Part 3: Sampling, data collection and analysis. Eur J Gen Pract. 2018;24:9–18. doi:10.1080/13814788.2017.1375091.

12. Willis N, Milanzi A, Mawodzeke M, Dziwa C, Armstrong A, Yekeye I, et al. Effectiveness of community adolescent treatment supporters (CATS) interventions in improving linkage and retention in care, adherence to ART and psychosocial well-being: A randomised trial among adolescents living with HIV in rural Zimbabwe. BMC Public Health. 2019;19:1–9.

13. Damulira C, Mukasa MN, Byansi W, Nabunya P, Kivumbi A, Namatovu P, et al. Examining the relationship of social support and family cohesion on ART adherence among HIV-positive adolescents in southern Uganda: baseline findings. Vulnerable Child Youth Stud. 2019;14:181–90. doi:10.1080/17450128.2019.1576960.

14. Malo VF, Ritchwood TD, Relf M V., Bekker LG. Does Type of Social Support Influence Medication Adherence Among South African Adolescents Living With HIV? A Quantitative Analysis of Pilot Data. J Assoc Nurses AIDS Care. 2022;33:492–8.

15. Brown WE, Malagala H, Bajunirwe F. Social support, gender and pill burden influence viral load suppression among HIV-infected adolescents and young adults in rural south-western Uganda. Vulnerable Child Youth Stud. 2021;16:86–97. doi:10.1080/17450128.2020.1842954.

16. Denison JA, Burke VM, Miti S, Nonyane BAS, Frimpong C, Merrill KG, et al. Project YES!

Youth Engaging for Success: A randomized controlled trial assessing the impact of a clinic-based peer mentoring program on viral suppression, adherence and internalized stigma among HIV-positive youth (15-24 years) in Ndola, Zambia. PLoS One. 2020;15:1–19. doi:10.1371/journal.pone.0230703.

17. Casale M, Boyes M, Pantelic M, Toska E, Cluver L. Suicidal thoughts and behaviour among South African adolescents living with HIV: Can social support buffer the impact of stigma? J Affect Disord. 2019;245 October 2018:82–90. doi:10.1016/j.jad.2018.10.102.

18. Brooks M, Burmen B, Olashore A, Gezmu AM, Molebatsi K, Tshume O, et al. Symptoms of depression, anxiety, and thoughts of suicide/self-injury in adolescents and young adults living with HIV in Botswana. African J AIDS Res. 2023;22:54–62.

19. Nanteza A, Gumikiriza-Onoria J, Santoro AF, Karungi C, Ferraris CM, Tsapalas D, et al. Reported Suicide Attempts among Adolescents in Uganda: Differences by HIV Status. AIDS Behav. 2024;:1004–10.

20. Olashore AA, Paruk S, Tshume O, Chiliza B. Depression and suicidal behavior among adolescents living with HIV in Botswana: a cross-sectional study. Child Adolesc Psychiatry Ment Health. 2022;16:1–8. doi:10.1186/s13034-022-00492-9.

21. Zhan S, Ouyang F, Zhai W, Yang H. Prevalence of mental disorders among young people living with HIV: a systematic review and meta-analysis. Front public Heal. 2024;12 August:1392872.

22. Grinstead OA, Van Der Straten A. Counsellors' perspectives on the experience of providing HIV counselling in Kenya and Tanzania: The voluntary HIV-1 counselling and testing efficacy study. AIDS Care - Psychol Socio-Medical Asp AIDS/HIV. 2000;12:625–42.

23. Grinstead OA. HIV counseling for behavior change. AIDS Educ Prev. 1997;9:125–32.
