# Supplementary material for: Crests, troughs, and plateaus: Using story theory to explore the experiences of adolescents and young people living with HIV in Kampala, Uganda
Source: PLoS One. 2026 Mar 25;21(3):e0345499. doi: 10.1371/journal.pone.0345499 (PMC13016292; doi:10.1371/journal.pone.0345499)
Supplement: Attachment — Submitted filename: Response to Reviewers.docx [file pone.0345499.s004.docx]

**Responses to Reviewers**

| **No** | **Reviewer comment** | **Response** |
| --- | --- | --- |
|  | **Reviewer 1** |  |
|  | There should have been more context to the lower Troughs in terms of duration of diagnosis, but it is just a suggestion. The article will also have benefited from some more explanatory note on whether double consent from both the parent/guardian and from the participants below 15, not just the guardian. The article covered important highlights but will benefit from more descriptive demographics of the environment. | Thank you for the suggestion to provide more context regarding the lower troughs and duration of diagnosis. This context is already included in the final sentence of the first paragraph of the Introduction, which discusses the challenges faced by young people living with HIV, including their struggle to cope with the diagnosis.  Regarding the issue of double consent, all participants under 18 provided assent. Additionally, as indicated in the ethics section, parents or guardians were requested to provide permission for their child to be involved in the study.  The comment regarding the demographics of the environment was unclear. However, the first sentence of the second paragraph in the Introduction and the first paragraph of the Results section already provide the relevant demographic information for this study |
|  | **Reviewer 2** |  |
|  | The writing is clear, concise, and easy read in standard English. All methodological processes have been presented in their common order. The theoretical framework conjoins well with the methodology, the analysis and discussions. Only few highlighted inconsistences need to be corrected. Also, ethical issues as indicated in the script comments | Thank you for the positive feedback on the coherence of the paper and for highlighting the inconsistencies. These inconsistencies have been corrected as indicated on page 6 and 7, and we have added a statement on page 6 indicating that counseling services were available in case any participant experienced emotional distress during the interview. |
|  | **Reviewer 3** |  |
|  | This is very interesting potentially impactful piece of work on HIV/AIDS, the work has great lessons that can be employed in health system provision at large. Briefly explain the story theory. | A brief explanation of the story theory has been included on page 4 |
| 1 | Use a checklist (such as the Consolidated criteria for reporting qualitative studies) to systematically report the qualitative findings | The consolidated criteria for reporting qualitative research (COREQ) was used in reporting the findings. |
| 2 | Research Team and reflexivity |  |
| a | Show experience that researchers have | The experience of the researchers has been highlighted on page 7 |
| b | Share reflexivity of the study. | A reflexivity statement has been included in the first paragraph on page 8. |
| c | How many participants refused to participate and how many dropped off? Any repeat interviews? | One participant withdrew from the study. No repeat interviews were conducted, and this has been noted in the Data Collection section on page 6. |
|  | Study design |  |
| a | Briefly show how the story theory was applied in the study | A description of how the theory was used to guide the development of the interview guide and interview process is provided on page 6. |
| b | How was the tool (? interview guide) piloted? | Information about how the interview guide was piloted has been included on page 6. |
| 4 | Data Collection, Analysis and findings |  |
| a | What did member checking (if any) show? | Member checking was not conducted, and this has been acknowledged on page 15. |
| b | How many audio recordings were in English and how many were in Luganda? | There were 11 English recordings and 4 Luganda recordings, and this has been noted on page 7 in the Data Management and Analysis section. |
| c | Who translated the Luganda audio recordings, if any? | The Luganda audios were translated by I.N and A.N who are fluent in English and Luganda |
| d | Who read the transcripts? | The transcripts were read by OCP, and this had been made clear on page 7 |
| e | What did field notes show? | The field notes were taken but were not included in the analysis. |
| f | Show “how many” (by saying most participants / generally most participants) supported the theme: refer to your discussion section where you did that very well. | Thank you for pointing this out and it has been corrected on page 9. |
